# Preparation and Chromaticity Control of CoTiO_3_/NiTiO_3_ Co-Coated TiO_2_ Composite Pigments

**DOI:** 10.3390/ma15041456

**Published:** 2022-02-16

**Authors:** Yuan Chen, Wei Guo, Yuan Huang, Ying Chang, Zhishun Wei, Jiuxin Jiang, Pascal Boulet, Marie-Christine Record

**Affiliations:** 1 Hubei Provincial Key Laboratory of Green Light Industrial Materials, Hubei University of Technology, Wuhan 430068, China; chen.yuan@hbut.edu.cn (Y.C.); cy0025@hbut.edu.cn (Y.C.); wei.zhishun@hbut.edu.cn (Z.W.); jiuxinjiang@hbut.edu.cn (J.J.); 2 Collaborative Innovation Center for Green Lightweight Materials and Processing, Hubei University of Technology, Wuhan 430068, China; 3 School of Materials and Chemical Engineering, Hubei University of Technology, Wuhan 430068, China; gw1072849743@gmail.com (W.G.); yuanhuang356@gmail.com (Y.H.); 4 New Materials and Green Manufacturing Talent Introduction and Innovation Demonstration Base, Wuhan 430068, China; pascal.boulet@univ-amu.fr; 5 MADIREL, CNRS, Faculty of Sciences, Aix-Marseille University, CEDEX 20, 13397 Marseille, France; 6 IM2NP, CNRS, Faculty of Sciences, Aix-Marseille University, CEDEX 20, 13397 Marseille, France

**Keywords:** inorganic pigment, core–shell microspheres, NiTiO_3_, CoTiO_3_, composite pigment

## Abstract

In this study, home-made amorphous TiO_2_ microspheres with good mono-dispersity and large numbers of mesopores on the surface were used as substrates. The intermediate microspheres were obtained by adding Co/Ni sources with different Co/Ni molar ratios in a water bath and making them react by water bath heating. By calcining the intermediate microspheres deposited on the TiO_2_ ones, a core–shell structured spherical CoTiO_3_/NiTiO_3_ inorganic composite pigment was prepared. The synthesized pigments were characterized by X-ray diffraction (XRD), Raman spectroscopy, field-emission scanning electron microscopy (FE-SEM), transmission electron microscopy (TEM), energy dispersive X-ray spectrometry (EDS), laser particle size (LPS) analysis and colorimetry. The results show that when the calcination temperature is 800 °C and the Co/Ni molar ratio is 0.5:0.5, the pigments consist of a TiO_2_ core and outer ilmenite CoTiO_3_/NiTiO_3_ shell. Moreover, the surface of the product microspheres is smooth, and the particles are of regular sphericity with a uniform particle size of about 1.8 μm. The colorimetric analysis from the samples calcined at 800 °C shows color changes from yellow-green to dark green as the Co/Ni molar ratio increases (0.1:0.9 to 0.9:0.1). A Co/Ni molar ratio that is too high or too low results in the formation of by-products such as Co_3_O_4_ or NiO, respectively, which adhere to the product surface and affect the chromaticity of the product. This work has enabled the chromatic modulation of yellow-green inorganic pigments, providing a solution for the preparation of spherical inorganic pigments that are more suitable for industrial inkjet printing.

## 1. Introduction

Owing to excellent weather resistance, thermal stability and high hiding power, inorganic pigments are widely used in ceramics, high temperature materials, inks, coating materials and plastics [1,2,3,4,5]. However, most inorganic pigments contain toxic elements such as heavy metals, making inorganic pigments not only costly, but also have adverse effects on human health and the environment, which severely restricts their development [6,7,8]. Therefore, the pigment industry necessitates the development of new inorganic pigments or the improvement of existing technology to eliminate or greatly reduce the harmful effects of toxic elements on human health in conventional inorganic pigments [9,10,11,12,13,14,15].

In recent years, cobalt-based green and nickel-based yellow pigments, such as CoTiO_3_ and NiTiO_3_ ilmenite, have been proposed as feasible alternatives to chromium-based green and lead chromium/lead antimony-based yellow pigments, respectively, due to their low toxicity, excellent performance in green/yellow color and corrosion resistance [9,16,17,18,19,20,21]. In the structure of CoTiO_3_ (or NiTiO_3_), both Co^2+^ (or Ni^2+^) and Ti^4+^ cations occupy alternating layers of octahedral sites, each layer being composed of a unique type of element, giving it good chemical stability. There are many methods for the preparation of CoTiO_3_ or NiTiO_3_ pigments, such as the solid-state reaction method [22,23], co-precipitation method [24,25], sol-gel method [26,27,28,29], thermal decomposition method [30], combustion method [31,32], etc. The traditional solid phase reaction method is easy to employ and does not introduce impurities, but the temperature required to synthesize pigments is high, generally exceeding 1000 °C, which often leads to severe sintering, increasing the particle size of the pigment and causing agglomeration [33,34]. Compared to the solid phase reaction method, the wet chemical method requires a lower reaction temperature but tends to introduce impurities, and the synthesized pigments are not homogeneous in particle size and also tend to agglomerate. The uneven particle size and poor sphericity of the pigments often result in poor flow of the pigment particles when used in inkjet printing technology, and the pigment particles often clog the printheads. Sekhar et al. [26] obtained CoTiO_3_ by calcining precursors prepared by the sol-gel method at 700 °C, and Wang et al. [17] synthesized NiTiO_3_ nano-yellow pigments by calcining organic polymer precursors of NiTiO_3_ at 600 °C and 800 °C. All these results indicate that CoTiO_3_ green pigments and NiTiO_3_ yellow pigments can be synthesized by wet chemical methods below 1000 °C.

In order to improve the dispersibility and coloring power of pigment particles, researchers have worked on the synthesis of nano- or micro-sized pigments [9,20,35]. The higher specific surface area and smaller particle size allow the pigment particles to achieve relatively good properties. In addition, the pigment particles often exhibit better optical properties due to their uniform particle size distribution and regular particle morphology [36]. As the technology for preparing TiO_2_ nano- or micro-sized microspheres is relatively mature, researchers often use them as substrates to synthesize CoTiO_3_ or NiTiO_3_ on their surface to prepare inorganic green or yellow composite pigments. Among them, Zou et al. [9] synthesized spherical CoTiO_3_@TiO_2_ green composite pigments using amorphous nano spherical TiO_2_ as a substrate at 800 °C, and He et al. [37] used a similar method to synthesize spherical NiTiO_3_@TiO_2_ yellow composite pigments.

A literature research reveals that there are many studies on the synthesis of either CoTiO_3_ or NiTiO_3_ on the surface of TiO_2_ to prepare composite pigment powders [9,20,37]. However, to our knowledge, there is no literature reporting on the co-synthesis of both CoTiO_3_ and NiTiO_3_ on the surface of TiO_2_. In this study, home-made amorphous TiO_2_ microspheres with good mono-dispersity and large numbers of mesopores on the surface were used as substrates. Intermediate microspheres were obtained by adding Co/Ni sources with different Co/Ni molar ratios in a water bath and making them react by water bath heating. Subsequently, composite pigments with CoTiO_3_/NiTiO_3_ co-coated on the TiO_2_ surface were prepared by calcination of the intermediate microspheres, and the modulation of the pigment particle morphology and chromaticity was achieved.

## 2. Materials and Methods

### 2.1. Materials

The raw materials were urea (CH_4_N_2_O, Sinopharm Chemical Reagent Co., Shanghai, China, >99.5%), anhydrous ethanol (C_2_H_5_OH, Sinopharm Chemical Reagent Co., Shanghai, China, >99.5%), Co(NO_3_)_2_·6H_2_O (Aldrich, Wuxi, China, ACS reagent), NiCl_2_·6H_2_O (Aldrich, Wuxi, China, ACS reagent) and tetrabutyl titanate (TBOT, Shanghai Macklin Biochemical Co., Shanghai, China, >99.5%). Solutions were prepared with deionized water (Molecular Lab water ultra-purifier, Shanghai, China).

The precursor TiO_2_ microspheres used in this study were homemade using a low-temperature modified Stöber method, which was previously reported by our group [38]. In this method, aqueous KCl and tetrabutyl titanate were added to ethanol and reacted at a low temperature of −10 °C for 5 h. The precursor was then collected by centrifugation, washed three times with alcohol and once with water, and subsequently freeze-dried, resulting in amorphous TiO_2_ microspheres.

### 2.2. Synthesis of NiTiO_3_/CoTiO_3_ Co-Coated TiO_2_ Composite Pigments

**Figure 1:**
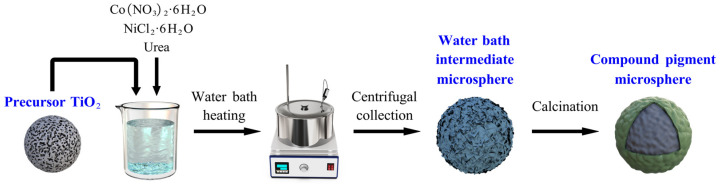
Process diagram for the preparation of composite pigment powders.

The preparation process of the composite pigment powders is shown in Figure 1.

The TiO_2_ microspheres prepared using the above method (0.3 g) were first ultrasonically dispersed in 100 mL of deionized water, and then Co(NO_3_)_2_·6H_2_O, NiCl_2_·6H_2_O and 12 g urea were added under magnetic stirring for 10 min. The molar ratios of Co/Ni/TiO_2_ were 0.1:0.9:1, 0.3:0.7:1, 0.5:0.5:1, 0.7:0.3:1, and 0.9:0.1:1. The above suspensions were subsequently heated in a water bath at 80 °C under magnetic stirring for 8 h to obtain intermediate microspheres according to the reaction equations:

Co^2+^ + 2OH^−^ → Co(OH)_2_, (1)

Ni^2+^ + 2OH^−^ → Ni(OH)_2_, (2)

The precipitate was then collected by centrifugation, washed three times with water, and dried at 50 °C for 12 h. The dried sample was calcined at 800 °C for 3 h. The corresponding reaction equations are as follows:

CoO + TiO_2_ → CoTiO_3_, (3)

NiO + TiO_2_ → NiTiO_3_, (4)

### 2.3. Characterization Techniques

The structural properties of the products were characterized by X-ray diffraction (XRD) using an X-ray diffractometer (PANalytical, Empyrean, Almelo, Netherlands) with Cu K*α* radiation (*λ* = 0.154 nm) and a 2*θ* scan range of 20°–75° at a rate of 2°/min. Raman spectra were recorded on a XploRA PLUS spectrometer (HORIBA, Lille, France) using a 532 nm wavelength laser source.

The morphologies and elemental ratio of the samples were determined by a field-emission scanning electron microscope (FE-SEM, SU8010, Hitachi, Tokyo, Japan) operating at 5 kV with an energy dispersive X-ray spectrometer (EDS, X-Max^N^, OXFORD, Oxford, Britain). TEM and HRTEM images were obtained using a transmission electron microscope (TEM, JEM-2100, JEOL, Tokyo, Japan) with an acceleration voltage of 200 kV.

A laser particle size (LPS) analyzer (Malvern MS 2000, Malvern, Britain) was employed to characterize the size distribution of the powders. The photographs of the synthesized composite pigments were obtained directly with a Canon m50 camera. The color tones of the pigments were evaluated by measuring L*, a*, and b* parameters with a colorimeter CR-10Plus (Konica Minolta, Tokyo, Japan), in which L* indicates the color lightness (from 0 (black) to 100 (white)), a* denotes the red/green intensity (negative and positive values correspond to green and red colors, respectively), and b* represents the yellow/blue intensity (negative and positive values correspond to blue and yellow colors, respectively).

## 3. Results

### 3.1. X-ray Diffraction

**Figure 2:**
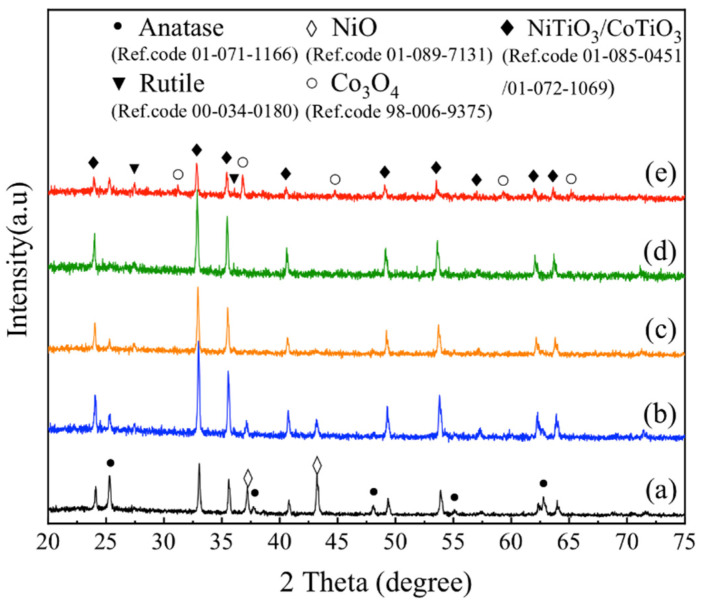
XRD patterns of the products after calcination at 800 °C for samples with different Co/Ni molar ratios: (**a**) 0.1:0.9, (**b**) 0.3:0.7, (**c**) 0.5:0.5, (**d**) 0.7:0.3, (**e**) 0.9:0.1.

Figure 2 shows the XRD patterns of the products after calcination at 800 °C for samples with different Co/Ni molar ratios. As shown in Figure 2a, when the Co/Ni molar ratio is 0.1:0.9, the products mainly consist of CoTiO_3_ and/or NiTiO_3_ (the diffraction peaks of CoTiO_3_ and NiTiO_3_ are very similar in position), NiO and anatase TiO_2_.

The presence of NiO diffraction peaks indicates that the reaction between NiO and TiO_2_ is not complete, and there is a surplus of NiO. This might be because the CoTiO_3_ and NiTiO_3_ produced by the reaction of the Co and Ni sources with TiO_2_, respectively, are coated on the surface of the TiO_2_ microspheres, preventing the further reaction of NiO with TiO_2_ and resulting in a surplus of NiO. As the amount of Co source increases, the NiO peak gradually disappears. When the Co/Ni molar ratio is increased to 0.9:0.1, as shown in Figure 2e, the diffraction pattern suggests the presence of Co_3_O_4_ phase in the product, which could similarly be due to the excess of Co source and the pre-formed CoTiO_3_ and NiTiO_3_ on the surface of the microspheres hindering the further reaction of CoO with TiO_2_. Further oxidation of the remaining CoO at high temperature results in the formation of Co_3_O_4_.

In addition, the results in Figure 2 show that the crystalline forms of TiO_2_ in the product are also influenced by the Co/Ni ratio. The home-made precursor TiO_2_ microspheres are amorphous [38], and when the Co/Ni ratio was low, the precursor TiO_2_ was converted to anatase phase in addition to reacting with Co and Ni sources; when the Co/Ni ratio was high, both anatase and rutile phases of TiO_2_ could be found in the products. The reason for this phenomenon could be that the presence of a Co source is more favorable to the formation of rutile phase TiO_2_, which also affects the brightness values of the products.

### 3.2. Raman Spectroscopy

**Figure 3:**
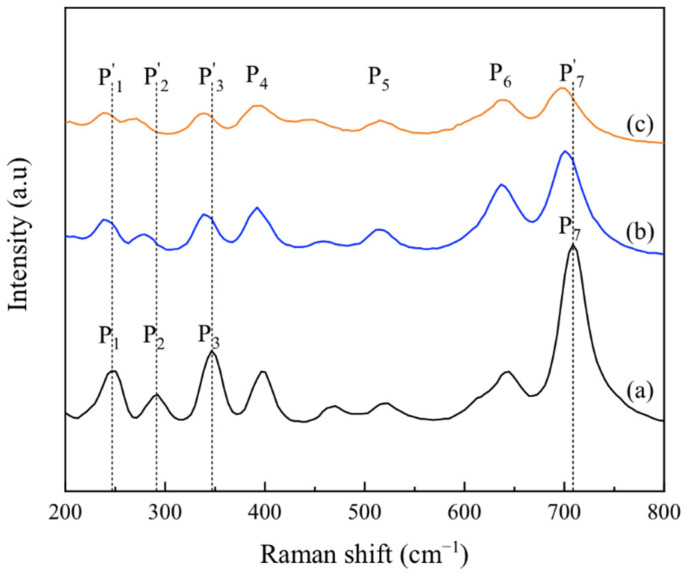
Raman spectra of products after calcination at 800 °C for samples with different Co/Ni ratios: (**a**) 0.1:0.9, (**b**) 0.5:0.5, (**c**) 0.9:0.1.

Because the diffraction peaks of CoTiO_3_ and NiTiO_3_ are very similar in position, making it difficult to distinguish between the two by X-ray diffraction, the structural properties and phase changes of the samples were further investigated by Raman spectroscopy. Figure 3a–c show the Raman spectra of the products after calcination at 800 °C for samples with Co/Ni molar ratios of 0.1:0.9, 0.5:0.5 and 0.9:0.1, respectively. Seven peaks from P1 to P7 can be observed in Figure 3a. A review of the literature shows that the characteristic peaks of anatase TiO_2_ are around 637.7, 515 and 396 cm^−1^ [39], corresponding to the three peaks at P4, P5 and P6 in Figure 3a. The characteristic peaks of NiTiO_3_ are around 238, 284, 338 and 705 cm^−1^, corresponding to the peaks at P1, P2, P3 and P7 in Figure 3a [40]. This indicates that when the Co/Ni molar ratio is 0.1:0.9, the product is dominated by NiTiO_3_. When the Co/Ni molar ratio is increased to 0.9:0.1, peaks P1, P2, P3 and P7 shift to P1′, P2′, P3′ and P7′ in Figure 3c, which correspond to those of CoTiO_3_ (the characteristic peaks of CoTiO_3_ are around 237, 266, 335 and 695 cm^−1^ [9]). It is worth mentioning that, in Figure 3a, the peak near 460 cm^−1^ corresponds to the characteristic peak of NiTiO_3_ [40], which can be explained by the fact that when the Co/Ni molar ratio is very low, and the phase in the product is dominated by NiTiO_3_. As the Co/Ni molar ratio increases, the peak shifts to near 445 cm^−1^ in Figure 3c, a position that corresponds to the characteristic peak of the rutile phase TiO_2_ [41]. This is in agreement with the XRD results in Figure 2, where the rutile phase appears in the product when the Co/Ni molar ratio is high, as seen in Figure 2e. The change in the position of the peaks between Figure 3a,c indicates that the product changes from being NiTiO_3_ dominated to CoTiO_3_ dominated as the Co/Ni molar ratio increases.

### 3.3. FE-SEM Observations

**Figure 4:**
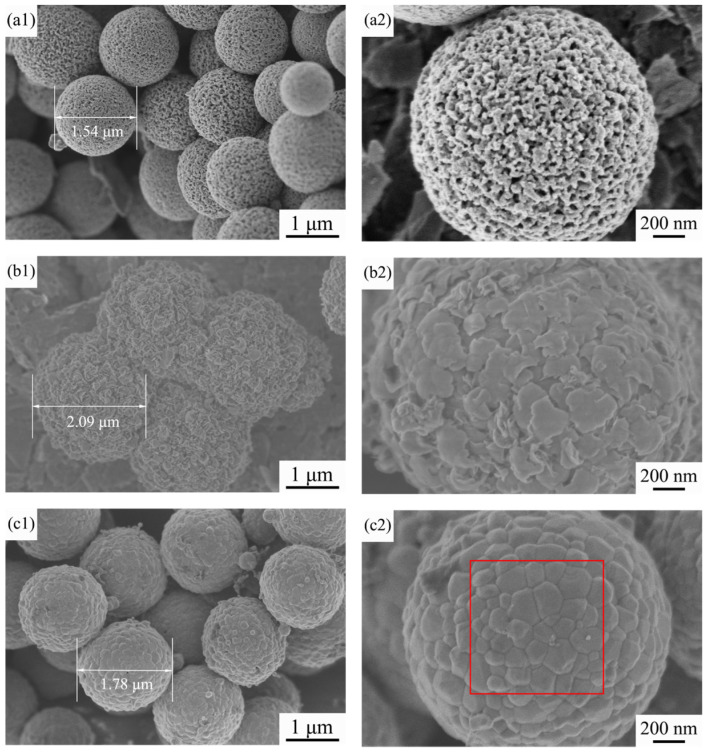
FE-SEM images of (**a1**,**a2**) the home-made TiO_2_ precursor microspheres, (**b1**,**b2**) the intermediate microspheres at a Co/Ni molar ratio of 0.5:0.5 and (**c1**,**c2**) the composite pigment powder obtained after calcination of the powder in (**b1**,**b2**) at 800 °C.

**Figure 5:**
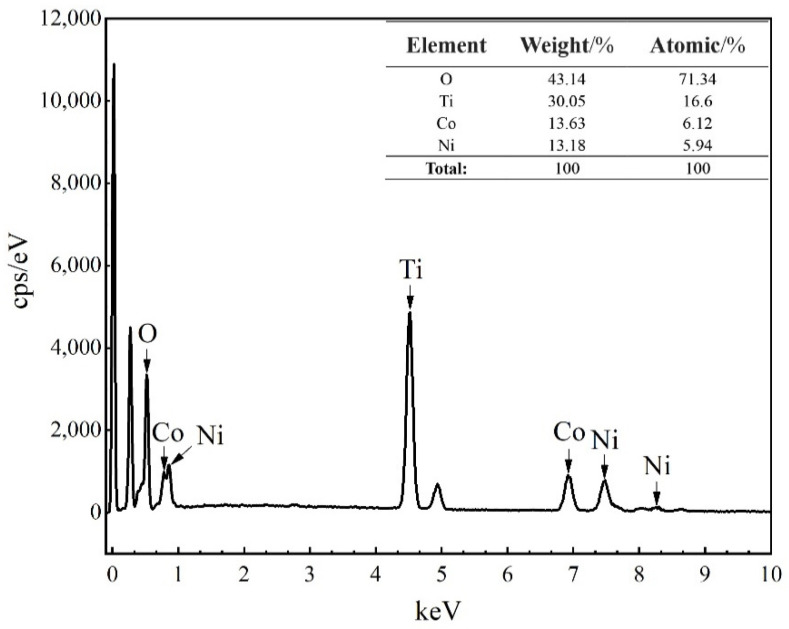
EDS spectrum of products calcined at 800 °C with a Co/Ni ratio of 0.5:0.5 (the analyzed area corresponds to the red box marked in Figure 4(c2)).

Figure 4 shows FE-SEM images of the precursor TiO_2_ microspheres, the intermediate microspheres at a Co/Ni molar ratio of 0.5:0.5 and the resulting composite pigment powder after calcination at 800 °C. The surface of the precursor TiO_2_ microspheres is mesoporous, the sphericity of the microspheres is regular and the particle size is relatively uniform, as can be seen in Figure 4(a1,a2). The detailed microscopic and structure characterization of the precursor TiO_2_ microspheres has already been reported by us [38]. Figure 4(b1,b2) shows many flakes attached to the surface of the intermediate microspheres, which are probably the reaction products of Co and Ni salts with urea during the water bath heating process (see Equations (1) and (2)). As the precursor TiO_2_ is a porous structured microsphere, and TiO_2_ tends to adsorb hydroxyl groups, this makes it easier for Co(OH)_2_ and Ni(OH)_2_ to be generated and attached to the TiO_2_ surface during the water bath process. Figure 4(c1,c2) shows that, after calcination, the flakes attached to the surface of the synthesized microspheres have disappeared due to the solid phase reaction between the oxides generated by the decomposition of Co(OH)_2_ and Ni(OH)_2_ at high temperatures and TiO_2_, which yields CoTiO_3_ and NiTiO_3_ on the surface of the synthesized microspheres (see Equations (3) and (4)), which agrees with the results of X-ray diffraction and Raman spectroscopy. For further proof, EDS analysis of the red boxed area in Figure 4(c2) was carried out to determine the elements of the product, and the results are shown in Figure 5. The spectrum confirms the presence of Ti, Ni, Co and O elements in the product, indicating that CoTiO_3_ and NiTiO_3_ were indeed generated on the surface of the synthesized microspheres. The estimated atomic ratios of these elements are close to the nominal ones. In contrast to the results of Zou et al. [9,20], the pigment particles prepared in this work have better mono-dispersity and a more homogeneous particle size while ensuring the purity of the sample, making them more suitable for industrial inkjet printing.

**Figure 6:**
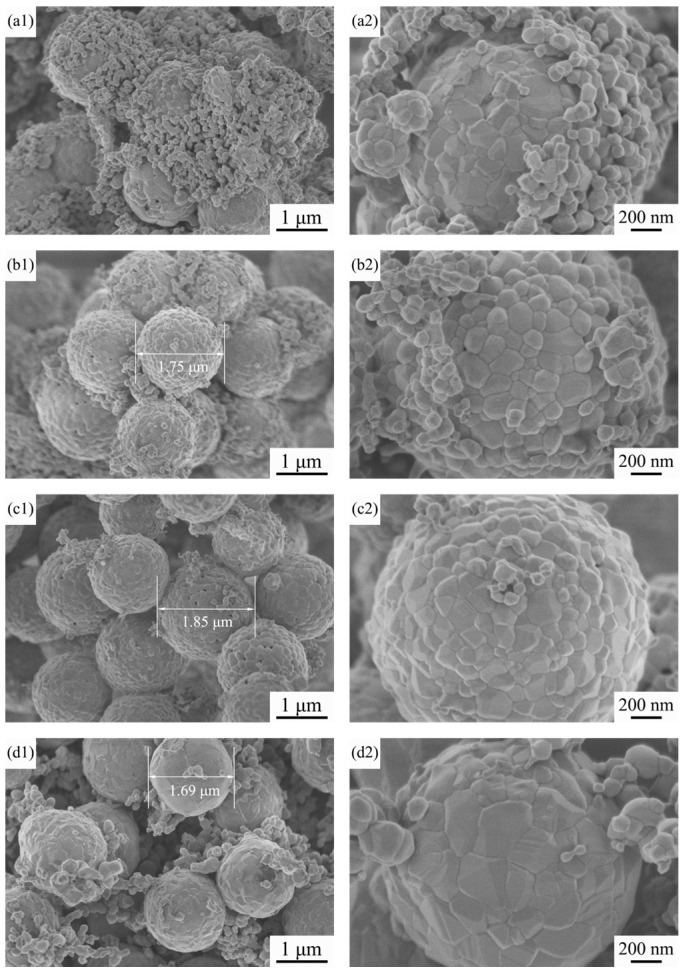
FE-SEM images of the intermediate microspheres after calcination at 800 °C for different Co/Ni molar ratios: (**a1**,**a2**) 0.1:0.9, (**b1**,**b2**) 0.3:0.7, (**c1**,**c2**) 0.7:0.3, (**d1**,**d2**) 0.9:0.1.

**Figure 7:**
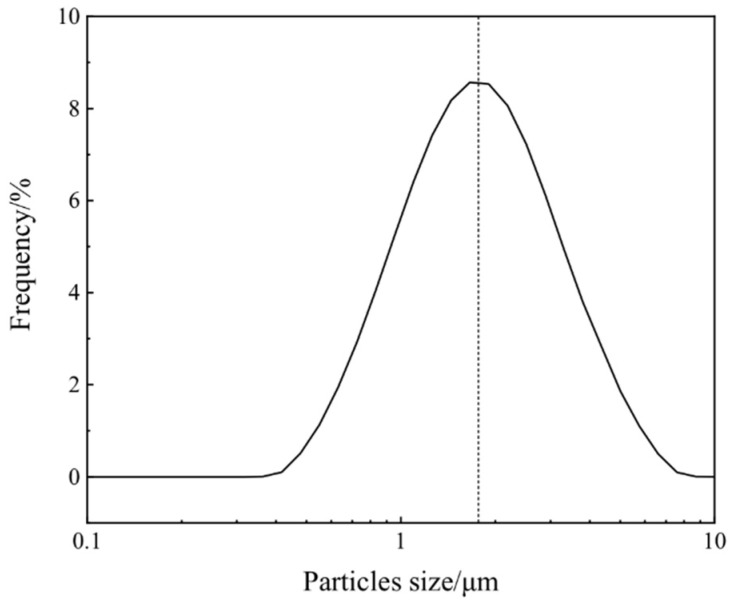
Particle size distribution of the composite pigment powder obtained after calcination at 800 °C for a Co/Ni molar ratio of 0.5:0.5.

Figure 6 shows the FE-SEM images of the samples with different Co/Ni molar ratios after calcination at 800 °C. As shown in Figure 6(a1,a2), at a Co/Ni molar ratio of 0.1:0.9, many particles approximately 100 μm in size are attached to the surface of the calcined product. Combined with the XRD results in Section 3.1, we can conclude that these particles are mainly NiO left over from the incomplete reaction with TiO_2_. From Figure 4(c1,c2) and Figure 6(b1,b2), it can be stated that: (i) the remaining particles on the surface of the synthesized microspheres gradually decrease in number as the amount of Co source increases, and (ii) when the Co/Ni molar ratio is 0.5:0.5, the surface of the product microspheres is the smoothest, and the synthesized particles have regular sphericity and are homogeneous in size. Laser particle size analysis of the sample with a Co/Ni molar ratio of 0.5:0.5 was carried out, and the results are shown in Figure 7. It can be seen that the average particle size of composite pigments is around 1.8 μm. With a further increase in the Co/Ni molar ratio at 0.7:0.3 and 0.9:0.1, Figure 6(c1,c2,d1,d2) indicate that the number of particles attached to the surface of the synthesized microspheres gradually increases again. When the Co/Ni molar ratio is 0.9:0.1, it can be seen in Figure 6(d1,d2) that, similar to the situation when the Co/Ni molar ratio is 0.1:0.9, the particles attached to the product surface are mainly Co_3_O_4_ formed by further oxidation of the remaining CoO that has not reacted completely with TiO_2_.

### 3.4. TEM Images

**Figure 8:**
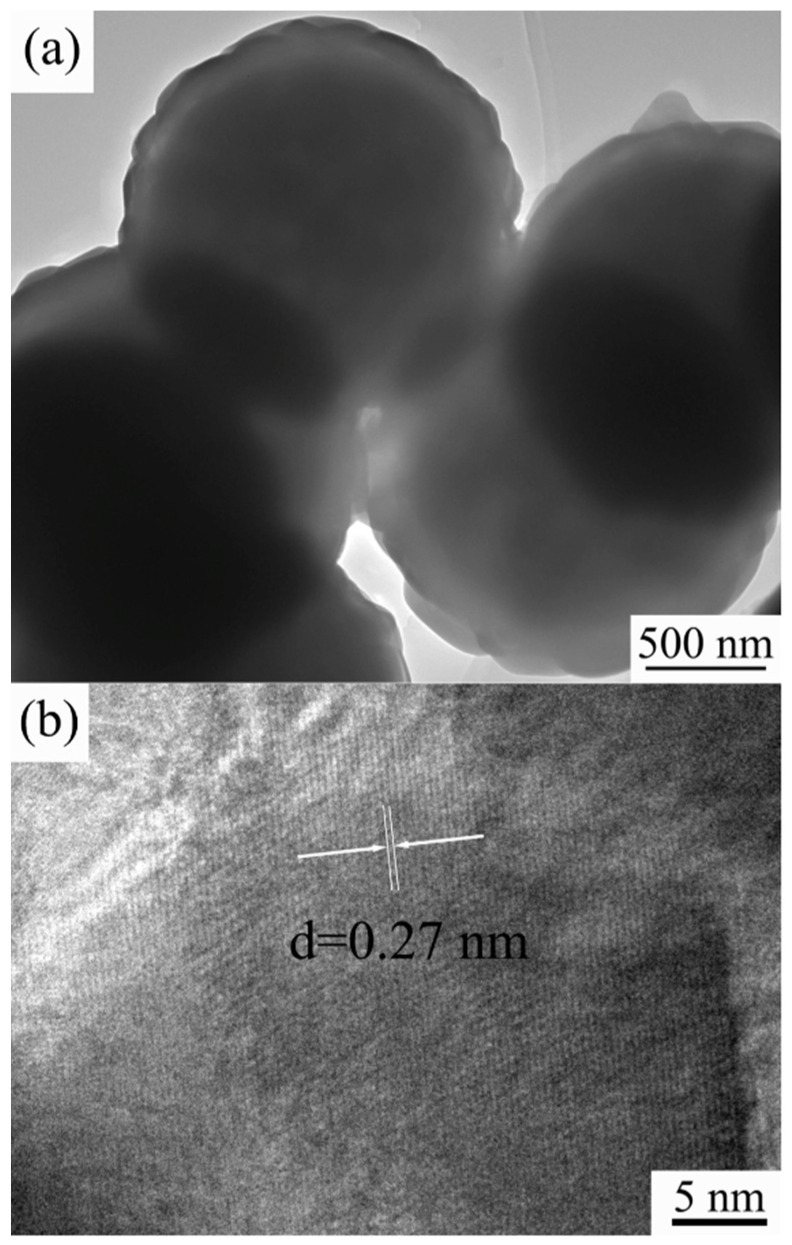
TEM (**a**) and HRTEM (**b**) images of the prepared composite pigments with a Co/Ni molar ratio of 0.5:0.5.

The microstructures of the obtained composite pigments with a Co/Ni molar ratio of 0.5:0.5 were further characterized by TEM, and the images are shown in Figure 8. The as-prepared pigments’ diameter was mainly around 1.8 μm (Figure 8a), which is consistent with the FE-SEM and laser particle size analysis results shown in Figure 4(c1,c2) and Figure 7, respectively. The composite pigment powder has a typical core–shell structure with a relatively smoothed out layer that is approximately 80 nm thick (Figure 8a). The high-resolution TEM (HRTEM) image of the particle edge provides information on the phase of the shell of the composite pigment. As shown in Figure 8b, the outer layer exhibits clear lattice fringes, which denotes a crystalline structure. The inter-planar spacing was calculated to be around 0.27 nm, which is consistent with the distance between two (104) crystal planes of ilmenite CoTiO_3_ and NiTiO_3_ [9,37]. By combining XRD and Raman results, we can conclude that when the Co/Ni ratio is 0.5:0.5, the prepared composite pigment particles consist of an outer CoTiO_3_/NiTiO_3_ shell and inner TiO_2_ core.

### 3.5. Colorimetric Analysis

**Figure 9:**
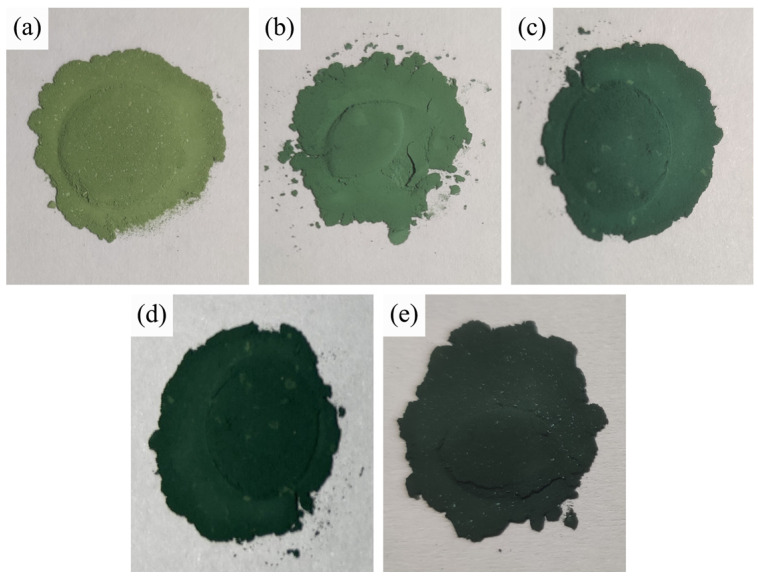
Photographs of the composite pigment powders obtained after calcination at 800 °C for samples with different Co/Ni molar ratios: (**a**) 0.1:0.9, (**b**) 0.3:0.7, (**c**) 0.5:0.5, (**d**) 0.7:0.3, (**e**) 0.9:0.1.

**Figure 10:**
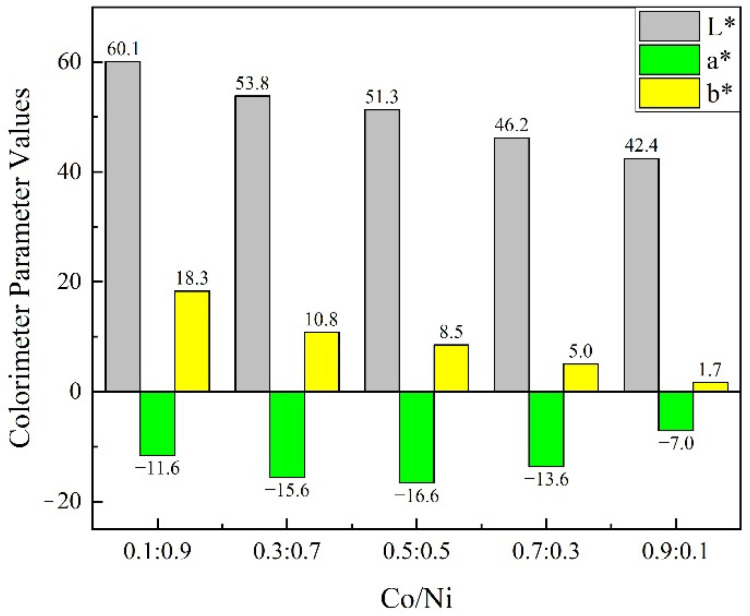
Colorimetric parameters of the composite pigment powders obtained after calcination at 800 °C for samples with different Co/Ni molar ratios: 0.1:0.9, 0.3:0.7, 0.5:0.5, 0.7:0.3, 0.9:0.1.

Figure 9 shows photographs of the composite pigment powders obtained after calcination at 800 °C for samples with different Co/Ni molar ratios. Figure 10 depicts the values of the colorimetric parameters of the corresponding composite pigment powders shown in Figure 9. As can be seen from Figure 9, the color of the calcined product changes from yellow-green to dark green as the Co/Ni molar ratio increases, and this trend is further confirmed by the values of the colorimetric parameters (Figure 10).

As seen from the results in Figure 10, the luminosity values (L*) of the calcined products decrease from 60.1 to 42.4 as the Co content increases. This might be due to the solid phase reaction of bright TiO_2_ with NiO and CoO to generate green CoTiO_3_ and yellow NiTiO_3,_ respectively, which act as a shell covering the surface of TiO_2_, resulting in a decrease in the luminosity values (L*) of the calcined products. Meanwhile, as the Co content increases, the excess black Co_3_O_4_ phase in the product shades the surface of the spherical powder, which leads to a decrease in the luminosity values (L*) of the calcined products [42].

The yellow values (b*) show a similar trend, decreasing from 18.3 to 1.7 as the amount of Ni source decreases, resulting in a decrease in the quantity of yellow NiTiO_3_ formed by the solid phase reaction with TiO_2_.

The green values (−a*) tend to increase and then decrease with the increase in Co content. The green values reach the maximum value of 16.6 when the Co/Ni molar ratio is 0.5:0.5, and then decrease. The reason for the initial increase in the green values is the increase in Co content, which causes TiO_2_ to react with more CoO in the solid phase to form more CoTiO_3_, which is green in color. As the Co content increases further, excess black Co_3_O_4_ phase is generated, which shades the CoTiO_3_ and leads to a decrease in the green values of the calcined products. The variation in the green values with the Co/Ni molar ratio for the samples prepared in this work follows a similar tendency to that observed by Zou et al. [9] with an increasing and then decreasing trend.

The colorimetric analysis shows that the change in phase composition leads to a difference in the color properties of the products, and the experimental results show that the chromaticity properties of the pigments can be modulated by changing the molar ratio of Co/Ni.

## 4. Conclusions

In this study, CoTiO_3_/NiTiO_3_ co-coated TiO_2_ core–shell structure composite pigment particles were prepared by wet chemical synthesis and high-temperature calcination using as substrates amorphous TiO_2_ microspheres with good mono-dispersity and a large number of mesopores on the surface. When the calcination temperature is 800 °C and the Co/Ni molar ratio is 0.5:0.5, the obtained product consists of a TiO_2_ core and outer ilmenite CoTiO_3_/NiTiO_3_ shell, and no impurities such as Co_3_O_4_ or NiO are found. The Raman spectroscopy technique has allowed us to identify the phases NiTiO_3_ and CoTiO_3_. The surface of the microspheres is smooth, and the particles are of regular sphericity, with a uniform particle size of about 1.8 μm. As the Co content increases, the luminosity (L*) and yellow (b*) values of the calcined products decrease (L* value: from 60.1 to 42.4, and b* value: from 18.3 to 1.7), and the green values (−a*) show increasing and then decreasing trends (−a* value: from 11.6 to 16.6 and then to 7.0). At the same time, the color of the product changes from yellow-green to dark green. A Co/Ni molar ratio that is too high or too low results in the formation of by-products such as Co_3_O_4_ or NiO, respectively, which adhere to the product’s surface and affect the chromaticity of the product. This work has enabled the chromatic modulation of yellow-green inorganic pigments, providing a solution for the preparation of spherical inorganic pigments that are more suitable for industrial inkjet printing.

## Data Availability

Not applicable.
